# Characterizing the temporal discrimination threshold in musician’s dystonia

**DOI:** 10.1038/s41598-022-18739-y

**Published:** 2022-09-02

**Authors:** Friederike Borngräber, Martina Hoffmann, Theresa Paulus, Johanna Junker, Tobias Bäumer, Eckart Altenmüller, Andrea A. Kühn, Alexander Schmidt

**Affiliations:** 1 Berlin Center for Musicians’ Medicine, Charité-Universitätsmedizin Berlin, Berlin, Germany; 2 Kurt Singer Institute for Music Physiology and Musicians’ Health, Hanns Eisler School of Music Berlin, Berlin, Germany; 3 Charité-Universitätsmedizin Berlin, Movement Disorder and Neuromodulation Unit, Department of Neurology, Berlin, Germany; 4 Berlin Institute of Health (BIH) at Charité-Universitätsmedizin Berlin, Berlin, Germany; 5 Charité-Universitätsmedizin, Department of Neurology, Berlin, Germany; 6 Department of Neurology, University of Lübeck, Lübeck, Germany; 7 Institute of Systems Motor Science, University of Lübeck, Lübeck, Germany; 8 Institute of Music Physiology and Musicians’ Medicine, Hanover University of Music, Drama and Media, Hanover, Germany

**Keywords:** Neuroscience, Sensory processing, Predictive markers, Genetic markers, Dystonia, Neurophysiology

## Abstract

The temporal discrimination threshold (TDT) has been established as a biomarker of impaired temporal processing and endophenotype in various forms of focal dystonia patients, such as cervical dystonia, writer’s cramp or blepharospasm. The role of TDT in musician’s dystonia (MD) in contrast is less clear with preceding studies reporting inconclusive results. We therefore compared TDT between MD patients, healthy musicians and non-musician controls using a previously described visual, tactile, and visual-tactile paradigm. Additionally, we compared TDT of the dystonic and non-dystonic hand and fingers in MD patients and further characterized the biomarker regarding its potential influencing factors, i.e. musical activity, disease variables, and personality profiles. Repeated measures ANOVA and additional Bayesian analyses revealed lower TDT in healthy musicians compared to non-musicians. However, TDTs in MD patients did not differ from both healthy musicians and non-musicians, although pairwise Bayesian t-tests indicated weak evidence for group differences in both comparisons. Analyses of dystonic and non-dystonic hands and fingers revealed no differences. While in healthy musicians, age of first instrumental practice negatively correlated with visual-tactile TDTs, TDTs in MD patients did not correlate with measures of musical activity, disease variables or personality profiles. In conclusion, TDTs in MD patients cannot reliably be distinguished from healthy musicians and non-musicians and are neither influenced by dystonic manifestation, musical activity, disease variables nor personality profiles. Unlike other isolated focal dystonias, TDT seems not to be a reliable biomarker in MD.

## Introduction

Musician’s dystonia (MD) is an isolated, focal, and task-specific dystonia affecting up to 1–2% of professional musicians. Patients suffer from a painless muscle incoordination and/or loss of voluntary motor control while playing the instrument^1,2^. Pathophysiological findings in MD and other types of focal dystonia include reduced inhibitory mechanisms, altered sensory perception and sensorimotor integration as well as maladaptive plasticity^3^. These changes are found in multiple brain regions, e.g. basal ganglia, thalamus, midbrain, cortex and cerebellum, which is why dystonia currently is seen as a network disease^4^.

Temporal aspects of somatosensory processing have drawn increasing interest as potential biomarkers in differential workup and pathophysiological understanding of movement disorders. One widely studied perceptual measurement is the temporal discrimination threshold (TDT), defined as the shortest interval at which two stimuli can be detected to be asynchronous^5^. It is a sensitive marker of aberrant sensory integration in basal ganglia and has been shown to be abnormal in different types of focal dystonia, e.g. writer’s cramp^6^, blepharospasm^7^ and cervical dystonia^8^. A comprehensive model for the neuronal circuits involving TDT comprises that sensory stimuli (visual, sensory or auditory) access the superior colliculus, a sensorimotor structure in the dorsal midbrain, important for rapid detection of environmental stimuli and attentional orienting^9,10^. These stimuli are then processed through a feed forward pathway to intralaminal nuclei of the thalamus, substantia nigra and basal ganglia allowing selection of salient events for on-going behaviour^11,12^.

The TDT has been proposed as a potential endophenotype (i.e. a hereditary biomarker that segregates with a disease without being symptom of it) in different forms of focal dystonia (i.e. cervical dystonia, writer’s cramp, blepharospasm and spasmodic dysphonia), as 78–97% of patients^5,8,13^ and 44–52% of unaffected first-degree relatives show abnormalities, suggesting an autosomal-dominant inheritance^5,8,14^. In line with this hypothesis, an enlargement of the putamen as well as reduced putaminal and superior collicular activity can be found coherent with an abnormal TDT in cervical dystonia patients and their healthy family members^5,14,15^. As up to one third of MD patients report a positive family history of autosomal-dominant inherited dystonia^16,17^, extensive studies have been initiated to unravel possible genetic causes in MD families. Whereas known monogenic causes of dystonia, i.e. TOR1A, THAP1 or GNAL, have been excluded as a major cause^17–19^, recent studies revealed RAB12 as a plausible candidate gene causing MD in 1.7% of patients^20^, and an intronic variant in the ARSG gene increasing the risk to develop MD to a factor of 4.33^21^. But still, the far greater portion of genetic predisposition in MD remains unclear. This might be explained by reduced penetrance [i.e. a number of gene mutation carriers will remain unaffected], a phenomenon well known in focal dystonia^22^. Also, healthy non-musical family members who carry candidate genes might suffer from MD if they played an instrument on a professional level. In both cases, endophenotypes such as TDT can help detecting gene mutation carriage in unaffected family members^5^.

Previous studies evaluated visual TDT measurements as a potential endophenotype in MD patients. Abnormal TDT values were found in only 20% of MD patients when healthy non-musicians were used as reference and in 45% of MD patients when compared to healthy musicians^23^. A more recent study compared MD patients (hand and larynx), focal non-musician dystonia patients (hand and larynx) and healthy controls^24^. Interestingly, TDT scores of non-musician dystonia patients differed from healthy controls, whereas MD patients did not show elevated TDT values compared to the control group. However, in this study healthy professional musicians and non-musicians were included in the control group and TDTs were only measured in the visual modality. Since timing abilities improve as a consequence of long-time musical training^25^, it might be fruitful to have separate control groups for musicians and non-musicians. Additionally, it would be interesting to assess visual and tactile stimuli as patients with focal task-specific hand dystonia have proven alterations in spatial and temporal sensory discrimination^26,27^.

The aim of our study was to replicate the results of earlier reports^23,24^ in an independent and well-defined sample of MD patients with focal hand dystonia and evaluate the reliability of TDT as a biomarker in MD patients. To control for the above-mentioned shortcomings, we (1) added both a healthy musician and healthy non-musician control group and (2) enlarged the design by comparing visual and tactile stimulation. In addition, we compared different TDT modalities in dystonic and non-dystonic hands and fingers of patients to further characterize the biomarker regarding its global vs. local utilization as well as potentially influencing factors e.g., musical activity variables and personality profiles.

## Methods

### Participants

A total of 60 participants were recruited to the study, including 20 patients with focal musician’s dystonia (MD) of the hand, 20 healthy professional musicians and 20 non-musician controls. Patients were recruited via the Berlin Center for Musicians’ Medicine at the Charité and the Institute of Music Physiology and Musicians’ Medicine at the Hanover University of Music, Drama and Media. Diagnosis of MD was established by two neurologists with expertise in movement disorders and musicians’ medicine (AS, EA). Of the patients, 18 had received at least one treatment with botulinum toxin. Eleven patients were still regularly treated with botulinum toxin. For patients still treated, average time since the last botulinum injection and study participation was 11.11 weeks (standard deviation (SD) = 5.93, range: 4–20 weeks).

The first control group of healthy professional musicians was recruited from orchestras, music schools and universities in Berlin. Data of 12 individuals from the second non-musician control group have been reported previously^28^. Additional eight non-musicians were recruited from hospital staff of the Charité. All healthy participants were neurologically examined to screen for dystonia or other movement disorders. As former studies showed age- and sex-related differences of TDT scoring^5,29^, both control groups were age- and sex-matched to the MD group. Healthy musicians were also matched by instrument to the MD patients.

Exclusion criteria for patients and controls were a history of other neurological diseases or psychiatric disorders, cognitive impairment, reduced visual acuity that could not be corrected to normal and visual field defects. Table 1 includes characteristics of the three groups. The study was approved by the local Ethics Board of the Charité (EA2/186/16) and conducted in conformity with the Declaration of Helsinki. All participants gave written informed consent prior to study participation.

**Table 1:** Characteristics of patients and controls.

Sample characteristics	Musician’s dystonia patients (n = 20)	Healthy musicians (n = 20)	Healthy non-musicians (n = 20)
Sex (male/female)	14/6	14/6	14/6
Age, years (mean ± SD)	44.25 ± 11.55	44.85 ± 12.66	43.55 ± 11.30
**Instrument group**			–
Woodwind instruments (n)	2	2	–
String instruments (n)	5	5	–
Brass instruments (n)	1	1	–
Plucking instruments (n)	3	3	–
Keyboard instruments (n)	8	8	–
Drums (n)	1	1	–
Age of first instrumental practice, y (mean ± SD)	7.40 ± 3.46	6.85 ± 2.72	–
Years of instrument playing, y (mean ± SD)	36.25 ± 12.20	38.00 ± 12.24	–
Accumulated practice time on the instrument, h (mean ± SD)	47,944 ± 19,298	41,479 ± 28,383	–
Age of onset of dystonia, y (mean ± SD)	34.05 ± 8.59	–	–
Duration of dystonia, y (mean ± SD)	10.45 ± 9.39	–	–

*y* years, *h* hours, *SD* standard deviation.

### TDT measurement

Measurement of TDT was performed as described previously^5^. TDTs were determined in three modalities: visual (VV), tactile (TT) and visual-tactile (VT). For the visual modality, pairs of flashlights were presented to participants seven degrees into the peripheral visual field. In the tactile modality, participants received pairs of non-painful electrical stimuli on the index and middle finger of one hand. Electrical stimuli were administered using square-wave stimulators (0.1 mA steps, pulse length 0.5 ms, 400 V, DS7A Digitimer; Digitimer Limited, Welwyn Garden City, UK). The individual sensory perception threshold was determined first. The stimulation intensity was doubled then and compared between fingers. In the mixed tactile-visual modality, participants received one visual and one tactile stimulus on the same body side. Stimuli were presented every 5 s. The first pair was presented synchronously; then the inter-stimulus interval increased in steps of 5 ms. Participants had to report verbally if they perceived stimuli synchronously or asynchronously. If three consecutive stimuli pairs were reported to be asynchronous, the run was terminated, and the first value taken as the discrimination threshold. TDT measurement was repeated four times per modality and body side. The median of the four runs was calculated for each modality and body side. The order of the tasks varied between participants.

In 19 MD patients with unilateral focal hand dystonia (14 men, 5 women, mean age ± SD: 44.21 ± 11.86), TDTs were compared between dystonic and non-dystonic hand. All three modalities, including visual TDT, were used in this analysis. Visual TDT were classified as dystonic/non-dystonic according to the side of the affected hand (right/left).

Additionally, we compared tactile and visual-tactile TDTs of dystonic and non-dystonic fingers in 16 MD patients with a unilateral disorder affecting individual fingers (11 men, 5 women; mean age ± SD: 43.38 ± 12.94). For instance, if the index or middle finger was dystonic, then finger four and five were measured as non-dystonic finger. If finger four or five was dystonic, the measurement of index and middle finger were considered non-dystonic. None of the patients had dystonia in the thumb, so the thumb was not measured for comparing between dystonic and non-dystonic fingers. Patients with dystonia of both hands or complex unilateral dystonia affecting all fingers had been excluded in the comparison between dystonic and non-dystonic hands and fingers.

### Musical activity variables

Information about musical activity were collected in MD patients and healthy musicians using structured personal interviews. We assessed the age of first instrumental practice and total years of instrument playing. In addition, we asked for weekly practice time across the age decades (i.e. until 10 years, 11–20 years, 21–30 years, 31–40 years etc.). Weekly practice time were combined with the total years of instrument playing to calculate accumulated practice time on the instrument.

### Personality profiles

Personality profiles were assessed in 18 MD patients (13 men, 5 women, mean age ± SD: 45.33 ± 11.53) to investigate the association with TDT measures. The revised German version of the Neuroticism Extraversion Openness Five-Factor Inventory^30^ (NEO-FFI) was used to assess personality profiles. The NEO-FFI is a self-report multidimensional personality inventory measuring five personality dimensions: neuroticism, extraversion, openness to experiences, agreeableness, and conscientiousness. Each of the dimensions is assessed by 12 items, scored on a 5-point Likert scale. Sum scores of each dimension were calculated by summing up the respective items.

### Statistical analysis

TDT data are given as mean values and standard deviations. Since most of the TDT variables were not normally distributed, a non-parametrical approach was adapted throughout. TDTs were analyzed using a repeated measures design for non-normal data from the package MANOVA.RM^31^, which allows to calculate robust test statistics. Wald-type statistics (WTS) combined with a permutation procedure for p-values were calculated to account for non-normally distributed data and small sample sizes. For post-hoc analysis of within factors (i.e. modality, hands, fingers) we conducted one-way repeated measure ANOVAs using the RM function of the MANOVA.RM package for pairwise comparisons of factor levels. For post-hoc comparisons of the between factor (i.e. group) we conducted pairwise comparisons of the different groups using the package GFD to calculate WTS combined with a permutation procedure for p-values^32^. Bonferroni correction was used to adjust p-values for multiple comparisons in post-hoc analysis. We additionally applied repeated measures Bayesian ANOVAs and calculated Bayes factors (BF) which allow to quantify the relative evidence that the data provide for the alternative (H_1_) or null hypothesis (H_0_)^33,34^. Bayesian Analyses were calculated using JASP^35^ (version 0.14.1) with default priors. We calculated inclusion Bayes factors (BF_incl_) which indicate the evidence for the inclusion of a particular effect calculated across matched models. For post-hoc analysis, pairwise comparisons using Bayesian t-tests were calculated and reported as BF_10_ and posterior odds. Posterior odds are corrected for multiple testing as implemented in JASP. BF_10_ are uncorrected and indicate the probability of the data under the H_1_ compared to the H_0_. A BF < 1 is considered as evidence for the null hypothesis with a BF between 1 and 1/3 indicating weak evidence, between 1/3 and 1/10 moderate evidence and a BF < 1/10 strong evidence. Accordingly, a BF > 1 is considered as evidence for the alternative hypothesis with a BF between 1 and 3 indicating weak evidence, between 3 and 10 moderate evidence and a BF > 10 strong evidence. A BF of 1 is considered no evidence for or against one hypothesis^33^. Note that we calculated parametric Bayesian ANOVAs, since non-parametric alternatives are currently not available.

To compare music activity variables between MD patients and healthy musicians, Mann–Whitney tests were calculated. Exploratory correlation analyses between TDTs, musical activity variables, clinical parameters and results of the NEO-FFI were conducted using Spearman rank correlations with 95% confidence intervals (95% CI) using the package correlation^36^. The significance level was set at p < 0.05. Analyses were performed using R^37^ (version 3.6.3).

## Results

### Temporal discrimination threshold

First, a repeated measures ANOVA for non-normally distributed data with group (MD patients, healthy musicians, healthy non-musicians) as between-factor and modality (visual, tactile, visual-tactile) and body side (left, right) as within-factor was conducted. Since the main effect of body side and the interactions involving body side were not significant (see Supplementary Table S1 and Supplementary Fig. S1 in the “Supplementary Material” for detailed results), body side was not further included in the analysis. Instead, the mean of the two body sides was calculated for each modality.

**Figure 1:**
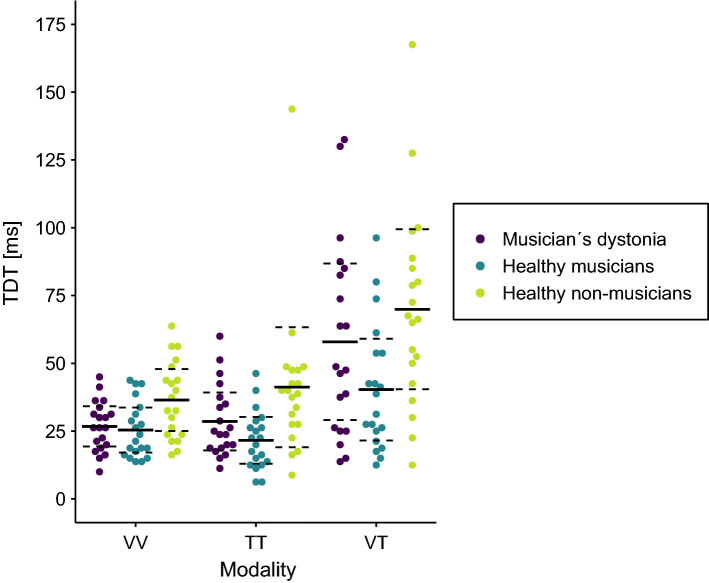
Visual (VV), tactile (TT) and visual-tactile (VT) temporal discrimination thresholds (TDT) in 20 patients with musician´s dystonia (purple), 20 healthy musicians (turquoise) and 20 healthy non-musicians (green). Solid lines represent the respective mean. Dashed lines indicate the 95% confidence interval of the mean.

Next, a repeated measures ANOVA for non-normally distributed data with modality (visual, tactile, visual-tactile, averaged across the two body sides) as within-factor and group (MD patients, healthy musicians, healthy non-musicians) as between-factor revealed a significant effect of group (WTS(2) = 13.07, p = 0.005). In line with this, the Bayesian ANOVA indicated strong evidence for the group effect (BF_incl_ = 13.29). Post-hoc analysis (with Bonferroni correction for overall 6 pairwise comparisons) did not show any differences of TDTs, averaged across the modalities, between MD patients (37.75 ms ± 16.94 ms) and healthy non-musicians (49.19 ms ± 22.84 ms; WTS(1) = 3.24, p = 0.47) as well as between MD patients and healthy musicians (29.10 ms ± 11.97 ms; WTS(1) = 3.47, p = 0.43). Pairwise Bayesian t-tests, however, revealed weak evidence for the alternative hypothesis in both comparisons (MD patients-healthy non-musicians: BF_10_ = 1.61, posterior odds = 0.94; MD patients-healthy musicians: BF_10_ = 1.41, posterior odds = 0.83). Healthy musicians had lower TDTs than healthy non-musicians (WTS(1) = 12.13, p = 0.005, BF_10_ > 100, posterior odds > 100). In addition, there was a significant effect of modality (WTS(2) = 66.89, p < 0.001) with Bayesian ANOVA also revealing strong evidence (BF_incl_ > 100). Post-hoc analysis revealed that, across the three groups, visual-tactile TDTs (56.06 ms ± 34.13 ms) were higher than both visual (29.52 ms ± 12.25 ms; WTS(1) = 48.68, p = 0.006, BF_10_ > 100, posterior odds > 100) and tactile TDTs (30.46 ms ± 20.03 ms; WTS(1) = 65.27, p = 0.006, BF_10_ > 100, posterior odds > 100). TDTs in the visual and tactile condition did not differ from each other (WTS(1) = 0.18, p = 1, BF_10_ = 0.15, posterior odds = 0.09). The interaction of group and modality did not reach significance, with the BF indicating only weak evidence for the null hypothesis, i.e. no presence of the interaction (WTS(4) = 9.22, p = 0.09, BF_incl_ = 0.44). Data of the three groups and modalities is plotted in Fig. 1.

### Comparison of dystonic and non-dystonic hands and fingers

To compare TDT values between dystonic and non-dystonic hands in MD patients we calculated a repeated measures ANOVA model with modality (visual, tactile, visual-tactile) and hand (dystonic, non-dystonic) as within factors. The main effect of modality was significant (WTS(2) = 20.14, p = 0.002). In line with this, Bayesian ANOVA revealed strong evidence for the effect of modality (BF_incl_ > 100). Post-hoc analysis (with Bonferroni correction for 3 pairwise comparisons) showed that across both hands visual-tactile TDTs (54.15 ms ± 36.09 ms) were higher than TDTs in the visual (26.58 ms ± 10.88 ms; WTS(1) = 17.86, p = 0.003, BF_10_ > 100, posterior odds > 100) and in the tactile condition (28.82 ms ± 17.88 ms; WTS(1) = 20.14, p = 0.003, BF_10_ > 100, posterior odds > 100). Visual and tactile TDTs did not differ from each other (WTS(1) = 0.95, p = 1, BF_10_ = 0.26, posterior odds = 0.15). No difference between dystonic (37.72 ms ± 28.06 ms) and non-dystonic hands (35.31 ms ± 26.03 ms) was observed (WTS(1) = 0.33, p = 0.579, BF_incl_ = 0.24). The interaction of modality and hand was not significant with the BF indicating only weak evidence for no presence of the interaction (WTS(2) = 5.72, p = 0.094, BF_incl_ = 0.47).

**Figure 2:**
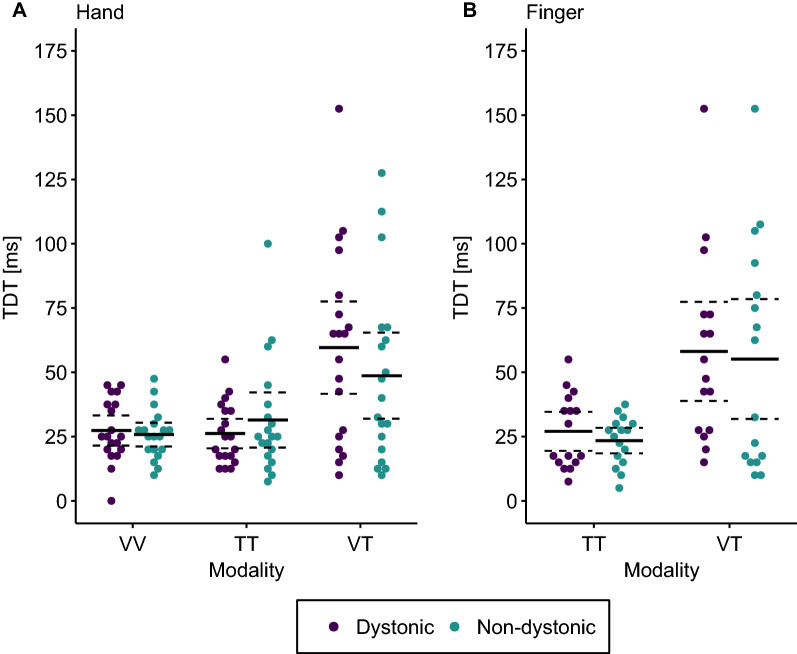
Temporal discrimination thresholds (TDTs) in dystonic and non-dystonic hands and fingers of patients with musician´s dystonia. (**A**) Comparison of visual (VV), tactile (TT) and visual-tactile (VT) TDTs in dystonic (purple) and non-dystonic (turquoise) hands in 19 patients with musician´s dystonia. (**B**) Comparison of tactile (TT) and visual-tactile (VT) TDTs in dystonic (purple) and non-dystonic (turquoise) fingers in 16 patients with musician’s dystonia. Solid lines represent the respective mean. Dashed lines indicate the 95% confidence interval of the mean.

We additionally compared TDT values between dystonic and non-dystonic fingers calculating a repeated measures ANOVA for non-normally distributed data with modality (tactile, visual-tactile) and finger (dystonic, non-dystonic) as within factors. There was a significant effect of modality (WTS(1) = 13.57, p = 0.002). Accordingly, the BF also indicated strong evidence for the inclusion of the modality effect (BF_incl_ > 100). Across the fingers, tactile TDTs (25.23 ms ± 12.04 ms) were lower than visual-tactile TDTs (56.64 ms ± 39.73 ms). There was no difference between dystonic (42.58 ms ± 31.44 ms) and non-dystonic fingers (39.30 ms ± 35.19 ms; WTS(1) = 0.46, p = 0.51, BF_incl_ = 0.28). The interaction between modality and finger was not significant with the BF indicating only weak evidence for no presence of the interaction (WTS(1) = 0.01, p = 0.93, BF_incl_ = 0.35). Results of the comparison between dystonic and non-dystonic hands and fingers are displayed in Fig. 2.

### Correlation of temporal discrimination thresholds with musical activity variables

MD patients and healthy musicians did not differ in the age of first instrumental practice (Mann–Whitney test: U = 190.00, p = 0.79), accumulated practice time on the instrument (U = 148.00, p = 0.16) or years of instrument playing (U = 210.50, p = 0.78). Descriptive statistics of the three musical activity variables are reported in Table 1.

We additionally examined relationships between TDTs and variables of musical activity. In healthy musicians, age of first instrumental practice correlated with visual-tactile TDT scores (r_s_ = 0.48, 95% CI [0.04, 0.77], p = 0.031), indicating that an earlier age of begin with musical training is associated with lower visual-tactile TDTs. In MD patients, however, earlier age of commencement of musical activity is associated with higher visual-tactile TDTs, although this correlation did not meet significance (r_s_ = -0.4, 95% CI [–0.72, 0.06], p = 0.078). Additionally, higher accumulated practice time is related to lower visual TDT in the patient group, although this correlation did not reach the significance level (r_s_− 0.41, 95% CI [–0.73, 0.05], p = 0.073). No correlation between accumulated practice time and TDT scores was observed in healthy musicians. Years of instrument playing was not associated with any of the TDT scores in both groups (see Table 2 for detailed results).

**Table 2:** Correlations between TDT scores and potentially influencing variables (musical activity variables, disease-related variables, NEO-FFI).

Group	Variable	TDT scores		
		VV	TT	VT
Healthy musicians (n = 20)	Age of first instrumental practice	r_s_ = 0.23 (95% CI [− 0.25, 0.62]), p = 0.33	r_s_ = 0.088 (95% CI [− 0.38, 0.52]), p = 0.71	r_s_ = 0.48 (95% CI [0.04, 0.77]), p = 0.031
	Years of instrument playing	r_s_ = 0.15 (95% CI [− 0.33, 0.56]), p = 0.54	r_s_ = 0.17 (95% CI [− 0.31, 0.58]), p = 0.48	r_s_ = 0.22 (95% CI [− 0.26, 0.62]), p = 0.34
	Accumulated practice time	r_s_ = 0.035 (95% CI [− 0.43, 0.48]), p = 0.89	r_s_ = 0.17 (95% CI [− 0.31, 0.58]), p = 0.48	r_s_ = 0.16 (95% CI [− 0.32, 0.57]), p = 0.50
Musician’s dystonia patients (n = 20)	Age of first instrumental practice	r_s_ = − 0.28 (95% CI [− 0.65, 0.20]), p = 0.23	r_s_ = − 0.027 (95% CI [− 0.47, 0.43]), p = 0.91	r_s_ = − 0.4 (95% CI [− 0.72, 0.06]), p = 0.078
	Years of instrument playing	r_s_ = − 0.22 (95% CI [− 0.61, 0.26]), p = 0.36	r_s_ = 0.13 (95% CI [− 0.34, 0.55]), p = 0.58	r_s_ = 0.12 (95% CI [− 0.35, 0.55]), p = 0.61
	Accumulated practice time	r_s_ = − 0.41 (95% CI [− 0.73, 0.05]), p = 0.073	r_s_ = − 0.11 (95% CI [− 0.53, 0.37]), p = 0.66	r_s_ = − 0.12 (95% CI [− 0.54, 0.35]), p = 0.62
	Disease duration	r_s_ = − 0.24 (95% CI [− 0.62, 0.24]), p = 0.32	r_s_ = 0.14 (95% CI [− 0.33, 0.56]), p = 0.55	r_s_ = 0.085 (95% CI [− 0.38, 0.52]), p = 0.72
	Age of dystonia onset	r_s_ = − 0.28 (95% CI [− 0.65, 0.20]), p = 0.24	r_s_ = − 0.26 (95% CI [− 0.64, 0.22]), p = 0.27	r_s_ = − 0.22 (95% CI [− 0.61, 0.26]), p = 0.35
	Time since last botulinum toxin treatment^a^	r_s_ = 0.043 (95% CI [− 0.65, 0.70]), p = 0.91	r_s_ = − 0.31 (95% CI [− 0.82, 0.46]), p = 0.41	r_s_ = 0.11 (95% CI [− 0.61, 0.73]), p = 0.77
	Neuroticism^b^	r_s_ = − 0.15 (95% CI [− 0.59, 0.36]), p = 0.56	r_s_ = − 0.16 (95% CI [− 0.60, 0.34]), p = 0.52	r_s_ = − 0.15 (95% CI [− 0.59, 0.35]), p = 0.56
	Extraversion^b^	r_s_ = 0.08 (95% CI [− 0.41, 0.54]), p = 0.75	r_s_ = 0.11 (95% CI [− 0.39, 0.56]), p = 0.66	r_s_ = − 0.19 (95% CI [− 0.61, 0.32]), p = 0.46
	Openness^b^	r_s_ = 0.16 (95% CI [− 0.34, 0.60]), p = 0.52	r_s_ = 0.041 (95% CI [− 0.45, 0.51]), p = 0.87	r_s_ = 0.066 (95% CI [− 0.43, 0.53]), p = 0.79
	Agreeableness^b^	r_s_ = 0.28 (95% CI [− 0.23, 0.67]), p = 0.27	r_s_ = 0.12 (95% CI [− 0.38, 0.57]), p = 0.63	r_s_ = 0.025 (95% CI [− 0.46, 0.50]), p = 0.92
	Conscientiousness^b^	r_s_ = − 0.19 (95% CI [− 0.62, 0.31]), p = 0.44	r_s_ = − 0.31 (95% CI [− 0.69, 0.20]), p = 0.21	r_s_ = − 0.13 (95% CI [− 0.57, 0.37]), p = 0.59

r_s_ = Spearman rank correlation coefficients. 95% confidence intervals in parentheses. Different TDT modalities are indicated as visual (VV), tactile (TT) and visual-tactile (VT).

^a^Data from 11 patients currently receiving treatment with botulinum toxin.

^b^Data from 18 patients.

### Correlations of temporal discrimination thresholds and disease-related variables

In MD patients, there were no correlations between disease duration and age of disease onset and any of the TDT measures. In 11 patients who were still treated with botulinum toxin at the time of study participation, average time since the last treatment did not correlate with the three TDT scores (see Table 2 for detailed results).

### Correlations of temporal discrimination thresholds with personality profiles

We additionally explored relationships of TDT scores with NEO-FFI results in 18 patients with MD. Correlation analysis did not show any significant correlations between any of the three TDT modalities and the five NEO-FFI sum scores, respectively (see Table 2 for results of the correlation analysis and Supplementary Table S2 for descriptive data of the NEO-FFI).

## Discussion

In line with previous observations^23^ healthy musicians had lower TDTs than non-musician controls, which, on an anatomical level can be explained by an enlargement of somatosensory and auditory representations due to long-lasting, extensive musical training^38^, resulting in better timing abilities irrespective of the sensory modality^25^. In contrast to the former study^23^, TDT values of our MD patients were not significantly different from both healthy musicians and non-musicians. Bayesian statistics, however, indicate weak evidence for the alternative hypothesis, i.e., differences between MD patients and healthy musicians as well as between patients and healthy non-musicians. Since the Bayes Factors in both comparisons are close to 1, these results rather indicate absence of evidence^34^ than evidence for group differences. These inconclusive results might be explained by the small sample size of our study. Also, in order to make our results comparable to previous studies, we applied the widely used staircase method instead of randomized stimuli presentation which might have contributed to a potential learning effect^12,39^. Additionally, we note a large variance of TDT values in our study, especially in the mixed visual-tactile task, thus making it difficult to detect differences between groups. A clear statement whether MD patients can reliably be distinguished from healthy musicians and non-musicians in terms of their TDT values therefore cannot be made.

Normal TDT levels in MD patients and healthy controls have also been shown in a former study^24^, in which groups, however, were more heterogeneous compared to our study, as they pooled laryngeal and focal hand dystonia together in the MD sample and professional musicians as well as non-musicians in the healthy control group. In this study, neural correlates of visual TDTs and brain activity were investigated using resting-state functional MRI^24^ in MD and non-musician focal dystonia patients as well as healthy (non-)musician controls. Whereas TDT values of MD patients did not differ from healthy controls, non-musician dystonia patients had significantly higher thresholds. In non-musician laryngeal and hand dystonia patients, an association, although not reaching significance, of TDT scores with lingual gyrus and cerebellar activation was found. In contrast, MD patients, show a distinctive pattern of correlations between TDT scores and brain activations (including the premotor, primary somatosensory, ventral extrastriate cortices, inferior occipital gyrus, precuneus and cerebellum). The authors concluded that by recruiting these different brain networks, MD patients seem to compensate for the lost neural correlates of TDT observed in healthy controls, which, in turn, could explain the normal TDT levels in patients^24^. A similar neural compensatory mechanism might have contributed to relatively normal TDT values in our MD patients, although we cannot prove this effect as we did not use neuroimaging methods. Also, in a TMS study comparing patterns of sensorimotor organization in the motor cortex in writer’s and musician's dystonia, neurophysiological differences with increased functional connectivity between muscle representations and subsequent loss of spatial specificity were found in MD patients, but not in writer’s dystonia^27^. Similarly, a study investigating neural correlates of different task-specific dystonia revealed decreased functional connectivity of the primary sensorimotor cortex, the parietal lobe and supplementary motor area in MD patients but not in non-musician’s dystonia, including writer’s cramp and spasmodic dysphonia^40^. These network changes suggest a weaker embedding of motor control and planning loops in MD but do presumably not affect TDT associated timing abilities.

As former TDT studies revealed no difference between the visual and tactile protocol, and the visual-tactile protocol seemed to have a high variability^5,13^, the visual protocol was solely used in further investigations, including studies with MD patients^23,24^. In contrast, we wanted to see whether the uni-modal visual task can be globally used as a biomarker of altered sensorimotor processing, or if tactile stimuli should be included to the analysis as patients with focal task-specific hand dystonia have proven alterations in spatial and temporal sensory discrimination^26,27^. Similar to the earlier results^5,13^, we found significantly higher and more variable TDTs in the visual-tactile compared to the uni-modal tasks (visual and tactile) for all three groups, which might be due to an activation of additional brain regions in cross-modal processing tasks^41^. Contrary to our expectations, we found no interaction of modality and group, indicating that there is no difference in visual and tactile temporal processing in MD patients. Furthermore, although musical training generally improved timing abilities in healthy participants, we saw no influence on a specific modality. This finding strengthens the global applicability of uni-modal TDT tasks.

Additionally, we compared visual, tactile and visual-tactile TDT of the dystonic and non-dystonic hand as well as dystonic and non-dystonic fingers as neurophysiological studies showed abnormal homuncular organization of the finger representation with reduced inter-digit separation, reversal and overlapping activation in the primary somatosensory cortex of patients with focal hand dystonia^42,43^. Clearly, we found no difference between dystonic and non-dystonic fingers, which might be partially explained by the fact that it can be difficult to separate dystonic (typically flexion of fingers) and compensatory movements (usually extension of fingers) in clinical practice^44^. Also, the impression of a determinable dystonic pattern of specific fingers might not be transferable to the underlying pathophysiology and both dystonic as well as compensatory movements are part of a complex motor pattern. In addition, we neither found a difference between the dystonic and non-dystonic hand, nor interaction of hand and modality. A recent study^45^ examined tactile space orientation evaluating distances between two touches across eight orientations on hands and forehead in different forms of isolated focal dystonia (cervical dystonia, blepharospasm and writer’s cramp) mirroring structural organization of somatosensory receptive fields. Also, the authors found no difference in affected and unaffected body parts^45^. Comprehensive electrophysiological testing of somatosensory inhibition and cortical plasticity in patients with basal ganglia lesion-induced acquired dystonia revealed no difference compared to healthy controls, questioning the presence of widespread abnormalities of somatosensory organization as a substantial pathophysiological feature^46^.

In our exploratory correlation analyses, we further investigated relationships between TDTs and its potentially influencing factors. In line with results of a previous study^24^, disease related variables as age of onset and disease duration had no effect on TDT scores. Although it long has been supposed that injections of botulinum toxin A only have a local effect on neuromuscular transmission of treated muscles, recent studies in other types of focal dystonia, however, show temporary alterations on cortical and subcortical level^47^. Since eleven patients were still treated with botulinum toxin A at the time of study participation, we tested the correlation of time since last botulinum toxin injection and TDT scoring. Similar to previous studies^5,15^, we did not find any relationship. In addition, another study compared TDT before and one month after botulinum toxin A injection in patients with cervical dystonia and did not find changes in TDT scoring^48^. It is therefore possible that the specific networks involved in TDT processing in MD are not affected by botulinum toxin therapy, although this remains speculative since, to our knowledge, there are no neurophysiological or neuroimaging studies investigating effects of chronic botulinum toxin treatment on the central nervous system in MD patients.

Furthermore, we analyzed to which extend musical activity influences different TDT modalities. Whereas duration of instrumental playing had no influence, the age of onset of instrumental practice correlated with the visual-tactile TDT in healthy musicians, indicating that a younger age of first practice is associated with a lower visual-tactile TDT. In early childhood neuronal plasticity is enhanced which is why early musical training enlarges sensory and association cortices, corpus callosum and auditory cortex improving visuomotor and auditory-motor synchrony^49,50^. In MD patients we see an opposite association, not reaching significance, towards a higher visual-tactile TDT in early trained patients which could be due to maladaptive plasticity with overlapping receptive fields^51^. Also, higher accumulated practice times seem to be associated with lower visual TDTs in patients, although this association did not meet significance. Longer hours of musical training might improve timing abilities and therefore also influence TDTs which is in line with a previous study showing that long-lasting musical training can improve timing abilities not only in auditory but also in visual domains^25^. However, it remains elusive why this association is not evident in tactile and visual-tactile stimuli or in the group of healthy musicians. The results of the correlation analyses should be interpreted with caution due to the low sample size of both musician´s dystonia patients and healthy musicians. To validate our exploratory correlation results and better estimate the strength of these effects, a bigger sample size would be needed.

It has been suggested that rather than sensory deficits of temporal processing, impaired decision-making might contribute to elevated TDT in cervical dystonia and that decision-making could be influenced by psychological comorbidities^52^. Previous studies reported psychological abnormalities in patients with MD. For instance, higher NEO-FFI neuroticism scores in female and higher openness scores in male MD patients compared to other isolated focal dystonias^53^ and higher neuroticism scores compared to both healthy musicians and non-musicians^54^ were found. As half of MD patients had signs of anxiety, perfectionism or stress in a former study, Ioannou and colleagues even postulated a new classification of ‘high psychological effect’ (HPE) MD and ‘low psychological effect’ (LPE) MD^55^. For the two subtypes, possible different pathophysiological paths were suggested: the LPE-MD might purely affect motor circuit, whereas the HPE-MD additionally involves emotional-memory and limbic networks of the cortical-basal ganglia-thalamic structures. In addition, the two subtypes should be considered in MD research as well as therapeutic management of patients^55^. To examine the relationship between personality profiles and TDT in our study, we correlated NEO-FFI and TDT scores and did not detect any correlations. However, data from patients with schizophrenia and major depression showed elevated acoustic TDTs compared to healthy controls, whereas dysthymic disorders seemed normal^56^. It therefore might be fruitful to investigate the relationship between TDT scores and psychological comorbidities (e.g., anxiety, depression) in MD patients in further studies.

In summary, we could replicate the results of earlier studies^23,24^ finding lower TDT in musicians compared to healthy non-musician controls. In contrast, TDTs in our MD cohort cannot reliably be distinguished from healthy musician and non-musician controls, which might be due to small sample sizes and high variability of TDT values. Furthermore, TDT values in MD patients were neither influenced by dystonic status, musical activity, disease variables nor personality profiles. Our results suggest that TDT therefore seems not to be a reliable biomarker of impaired sensory processing in MD and might not be a useful endophenotype in clinical assessment of MD patients and their relatives.

## Supplementary Information

Supplementary Information.

## Data Availability

Anonymized data of the study are available from the corresponding author upon reasonable request of qualified investigators.
