# Supplementary material for: Characterizing the temporal discrimination threshold in musician’s dystonia
Source: Sci Rep. 2022 Sep 2;12:14939. doi: 10.1038/s41598-022-18739-y (PMC9440005; doi:10.1038/s41598-022-18739-y)
Supplement: Supplementary file 1 — Supplementary Information. [file 41598_2022_18739_MOESM1_ESM.docx]

**Supplementary Materials:**

**Supplementary Table S1** Results of the ANOVA of group, modality and body side

| Effect | WTS | df | p | BF_incl_ |
| --- | --- | --- | --- | --- |
| Group | 13.07 | 2 | 0.005 | 13.11 |
| Modality | 66.89 | 2 | <0.001 | >100 |
| Group x modality | 9.22 | 4 | 0.088 | 1.02 |
| Body side | 0.23 | 1 | 0.633 | 0.14 |
| Group x body side | 4.41 | 2 | 0.127 | 0.25 |
| Modality x body side | 0.662 | 2 | 0.729 | 0.06 |
| Group x modality x body side | 0.381 | 4 | 0.987 | 0.04 |

WTS = Wald-type statistics; df = degree of freedom; BF_incl_ = inclusion Bayes Factor

**Supplementary Table S2** NEO-FFI scores of patients with musician’s dystonia^a^

| NEO-FFI score | Sum score  (mean ± SD) | Range of sum score (Min; Max) | Z-score  (mean ± SD) | Range of Z-score  (Min; Max) |
| --- | --- | --- | --- | --- |
| Neuroticism | 22.45 ± 7.2 | 8.73; 35.00 | 0.33 ± 0.94 | -1.69; 1.69 |
| Extraversion | 25.21 ± 4.95 | 16.00; 32.73 | -0.26 ± 0.76 | -1.85; 0.89 |
| Openness | 29.77 ± 5.47 | 22.91; 40.36 | -0.08 ± 0.93 | -1.19; 1.88 |
| Agreeableness | 31.33 ± 6.35 | 17.00; 39.27 | 0.28 ± 1.12 | -2.71; 1.52 |
| Conscientiousness | 33.08 ± 5.71 | 25.00; 45.00 | 0.12 ± 0.91 | -1,44; 1.98 |

SD = standard deviation; Z-scores were normalized for age and sex ^28^

^a^ Data from 18 patients (13 men, 5 women)


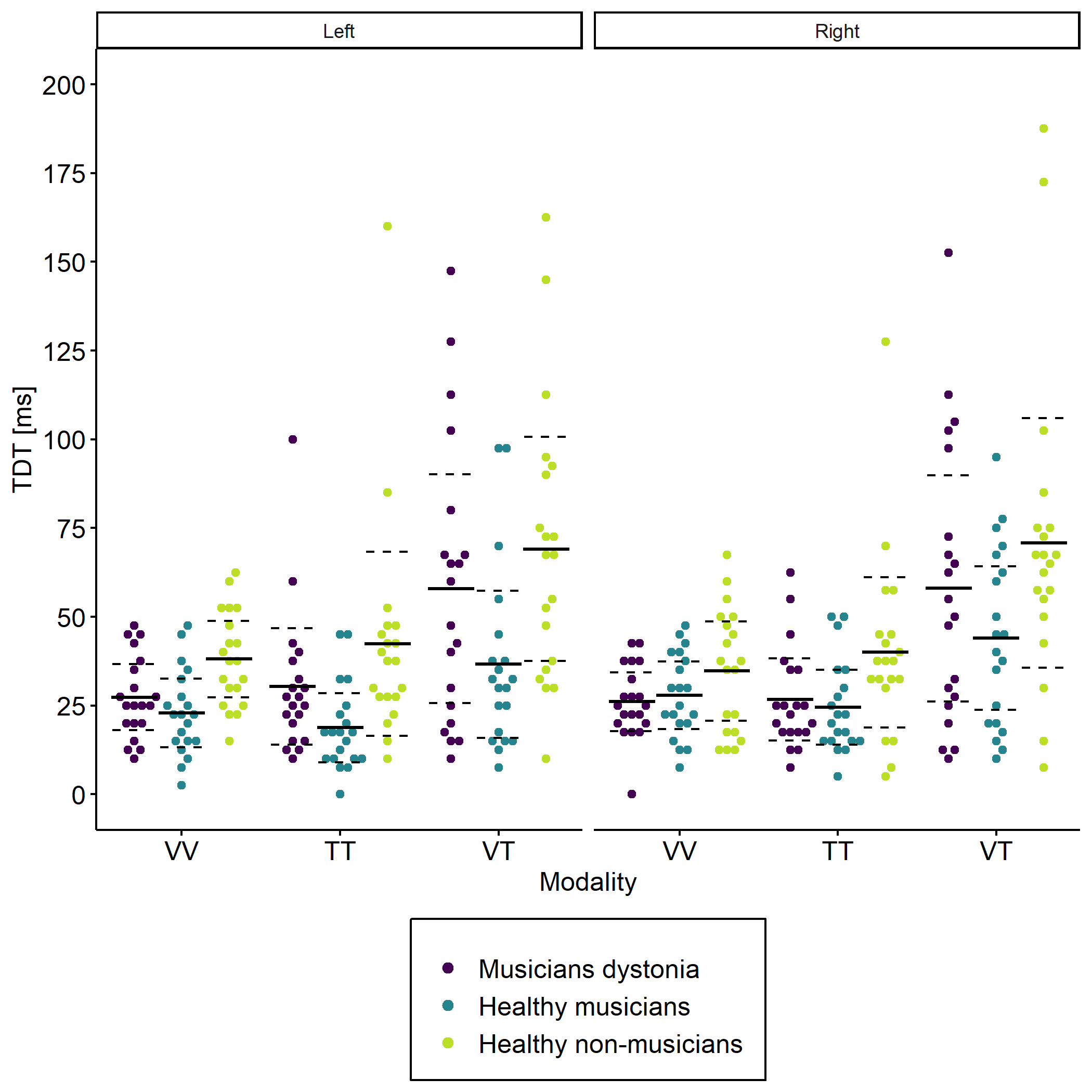


**Supplementary Figure S1.** Visual (VV), tactile (TT) and visual-tactile (VT) temporal discrimination thresholds (TDT) of the left and right body side in 20 patients with musician´s dystonia (purple), 20 healthy musicians (turquoise) and 20 healthy non-musicians (green). Solid lines represent the respective mean. Dashed lines indicate the 95% confidence interval of the mean.
